# Decontamination Using a Desiccant with Air Powder Abrasion Followed by Biphasic Calcium Sulfate Grafting: A New Treatment for Peri-Implantitis

**DOI:** 10.1155/2015/474839

**Published:** 2015-04-27

**Authors:** Giorgio Lombardo, Giovanni Corrocher, Angela Rovera, Jacopo Pighi, Mauro Marincola, Jeffrey Lehrberg, Pier Francesco Nocini

**Affiliations:** ^1^Clinic of Dentistry and Maxillofacial Surgery, Policlinico G.B. Rossi, University of Verona, Piazzale L.A. Scuro 10, 37134 Verona, Italy; ^2^Department of Dental Medicine, University of Cartagena, Avenida del Consulado # Calle 30 No. 48–152, Cartagena, Bolívar 130011, Colombia; ^3^Department of Biomaterials, Implant Dentistry Centre, 501 Arborway, Jamaica Plain, Boston, MA 02130, USA

## Abstract

Peri-implantitis is characterized by inflammation and crestal bone loss in the tissues surrounding implants. Contamination by deleterious bacteria in the peri-implant microenvironment is believed to be a major factor in the etiology of peri-implantitis. Prior to any therapeutic regenerative treatment, adequate decontamination of the peri-implant microenvironment must occur. Herein we present a novel approach to the treatment of peri-implantitis that incorporates the use of a topical desiccant (HYBENX), along with air powder abrasives as a means of decontamination, followed by the application of biphasic calcium sulfate combined with inorganic bovine bone material to augment the intrabony defect. We highlight the case of a 62-year-old man presenting peri-implantitis at two neighboring implants in positions 12 and 13, who underwent access flap surgery, followed by our procedure. After an uneventful 2-year healing period, both implants showed an absence of bleeding on probing, near complete regeneration of the missing bone, probing pocket depth reduction, and clinical attachment gain. While we observed a slight mucosal recession, there was no reduction in keratinized tissue. Based on the results described within, we conclude that the use of HYBENX and air powder abrasives, followed by bone defect grafting, represents a viable option in the treatment of peri-implantitis.

## 1. Introduction

Peri-implantitis is a condition that affects the mucosa and bone surrounding dental implants and is characterized by crestal bone loss [1, 2]. Depending on the qualification criteria, the incidence of peri-implantitis ranges from 18.8% to 51.9% [3–5]. It is generally accepted that the colonization of deleterious bacteria in the peri-implant microenvironment plays a major role in the etiology of peri-implantitis [1, 6–8]. Polymicrobial communities that colonize different microhabitats within the mouth are referred to as a biofilm [9, 10]. Similar to periodontitis, treatment modalities aimed at correcting peri-implantitis include the reduction or elimination of deleterious bacteria within the biofilms that coat the oral surfaces [11, 12].

Removal of oral biofilms, or the decontamination of the peri-implant microenvironment in patients suffering from peri-implantitis, can be achieved through both surgical and nonsurgical means [11, 12]. Proposed treatments for the decontamination of microenvironments during peri-implantitis include antibiotic and antiseptic treatment (both local and systemic), mechanical debridement, and laser treatment [13]. Surgical techniques are also used in the treatment of peri-implantitis, with the rationale that surgery grants increased access to the spaces inhabited by deleterious bacteria [13, 14]. However, strong evidence supporting any particular therapy in the treatment of peri-implantitis has yet to be established [13, 14]. In light of this, we attempted to treat a case of peri-implantitis using a topical desiccant (HYBENX Oral Tissue Decontaminant, EPIEN Medical, Inc.) as an adjunct to air powder abrasion (Airflow, EMS), followed by the application of an inorganic bone composite comprised of a mixture of bovine bone material (Bio-Oss, Geistlich Biomaterials) mixed with biphasic calcium sulfate (BondBone, MIS Implants Technologies Ltd.). HYBENX is an extremely hygroscopic solution that theoretically functions by denaturing the attachment proteins used by bacteria to adhere to the implant surface. This allows the more efficient subsequent removal of biofilm microbes. To our knowledge, no study using a topical desiccant as an adjunct to air powder abrasives has been published to date.

Here we report the treatment protocol and two-year follow-up of a 62-year-old patient who was treated using our novel surgical protocol for peri-implantitis. Based on the successful outcome our patient exhibited, we conclude that the use of a HYBENX as an adjunct to air powder abrasion, followed by composite bone grafting using inorganic bovine bone mixed with biphasic calcium sulfate, represents a possible future treatment for peri-implantitis that warrants further consideration and study.

## 2. Case Report

**Figure 1:**
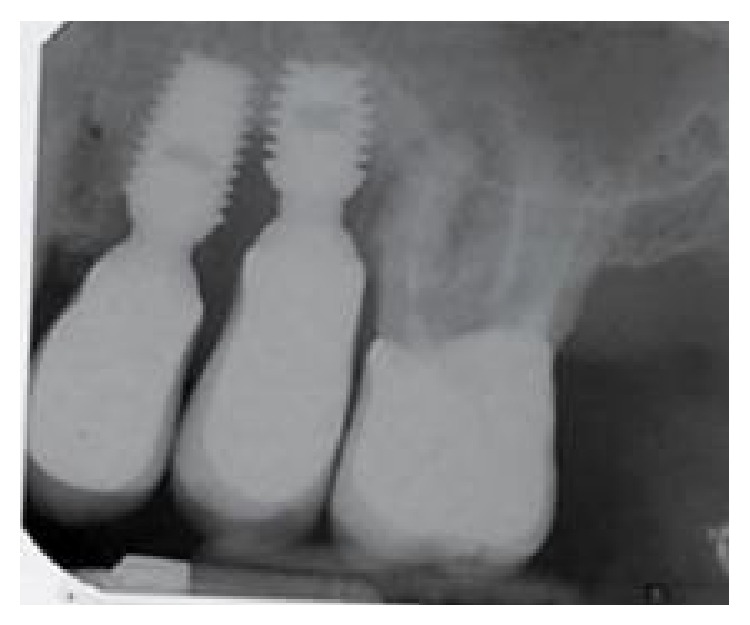
The baseline periapical radiograph indicated a deep interproximal peri-implant bone lesion.

**Figure 2:**
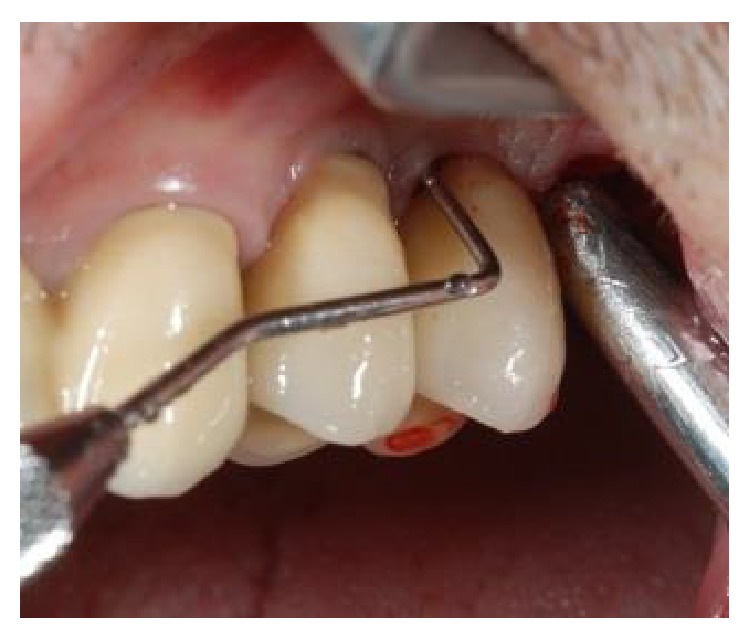
Initial probing revealed a deep peri-implant pocket between 2 short, single-crown locking taper implants, in positions 12 and 13.

A 62-year-old man reported discomfort and bleeding that occurred while brushing around his two single crown implants (upper left premolars: tooth numbers 12 and 13). Periapical radiographs revealed a large apical lesion adjacent to the neighboring molar and a bone loss pattern suggestive of peri-implantitis around the indicated implants (Figure 1). Probing pocket depths ranged from 7 to 9 mm (Figure 2). Initial treatment included the nonsurgical mechanical debridement of implants using ultrasonic devices and reinstruction in oral hygiene techniques. This initial treatment unfortunately resulted in insignificant reductions of pocket depths and inflammation.

Following the initial nonsurgical treatment, the option of removing the prosthesis to allow submerged healing was presented to and declined by the patient. After discussing the inherent risks involved, the patient gave written consent and agreed to proceed with open debridement and decontamination, followed by guided bone regeneration using bone composite [15].

In order to minimize clinical signs of inflammation, a single course of low abrasive air powder (Airflow) was implemented two weeks before surgery.

The surgical procedure is described as follows: briefly, after local infiltration of a 2% lidocaine solution with 1 mcg/mL epinephrine, sulcular incisions were made on the buccal and lingual/palatal side in an effort to preserve soft tissue. Full thickness flaps were then elevated with a periosteal release to allow for adequate flap mobilization and coronal advancement at the time of closure.

**Figure 3:**
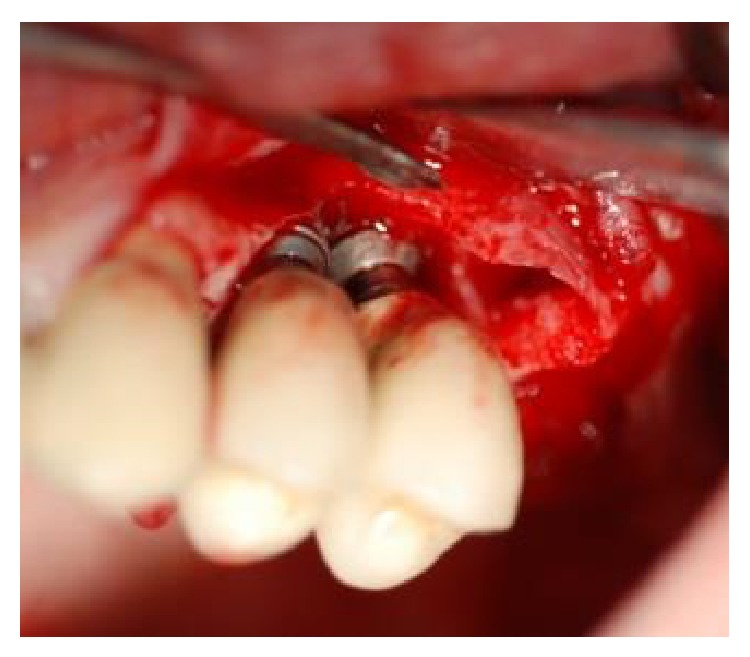
After elevation of the vestibular and palatal full thickness flaps, a crater-like defect characterized by interproximal bone loss was observed around the implants.

**Figure 4:**
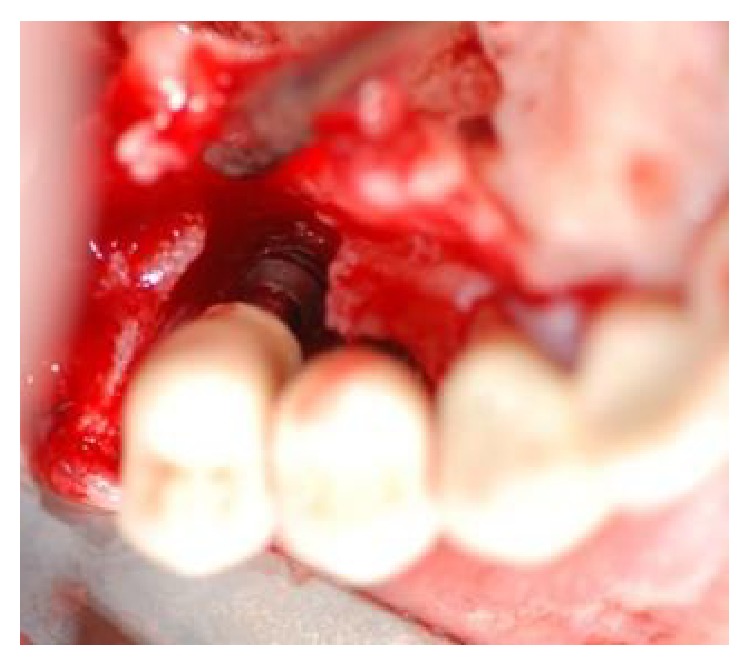
After elevation of the vestibular and palatal full thickness flaps, a crater-like defect characterized by interproximal bone loss was observed around the implants.

**Figure 5:**
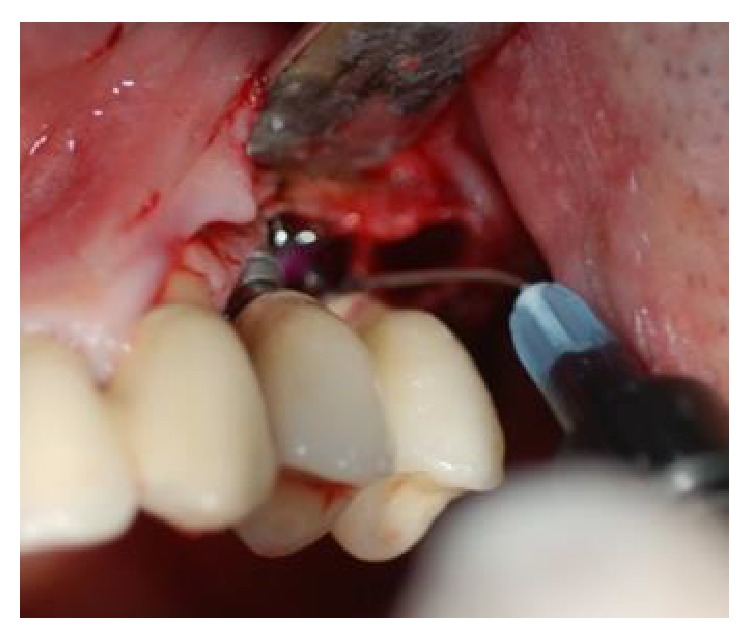
HYBENX was administered on the implant surface for 60 seconds and then thoroughly rinsed away with saline.

**Figure 6:**
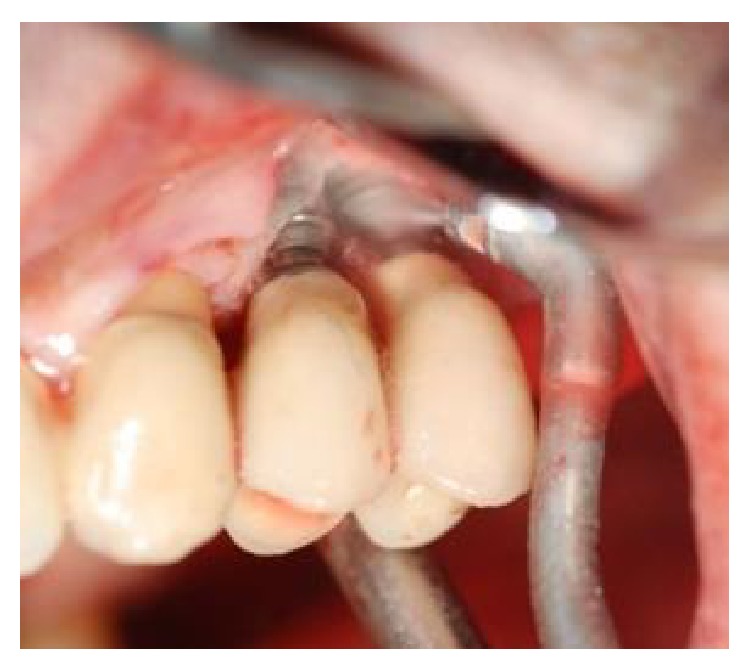
Debridement of the implant surfaces using air powder abrasion for 60 seconds.

Interproximal tissue was then removed and—after a thorough degranulation of the osseous defects—surgical exposure of the coronal portion of the implants was provided. Smoothening of buccally and supracrestally exposed implant parts was performed with the use of rotating burs (i.e., implantoplasty) (Figures 3 and 4) [16, 17]. The surface decontamination procedure consisted of a 3-step protocol that was repeated twice:

1. Application of HYBENX to the defect and implant surface, with 60-second incubation period (Figure 5).
2. Abundant irrigation of the defect with saline solution.
3. Administration of sodium bicarbonate-based abrasive air powder treatment (Airflow) to all contaminated and exposed parts of the implant surface for 60 seconds (Figure 6).

**Figure 7:**
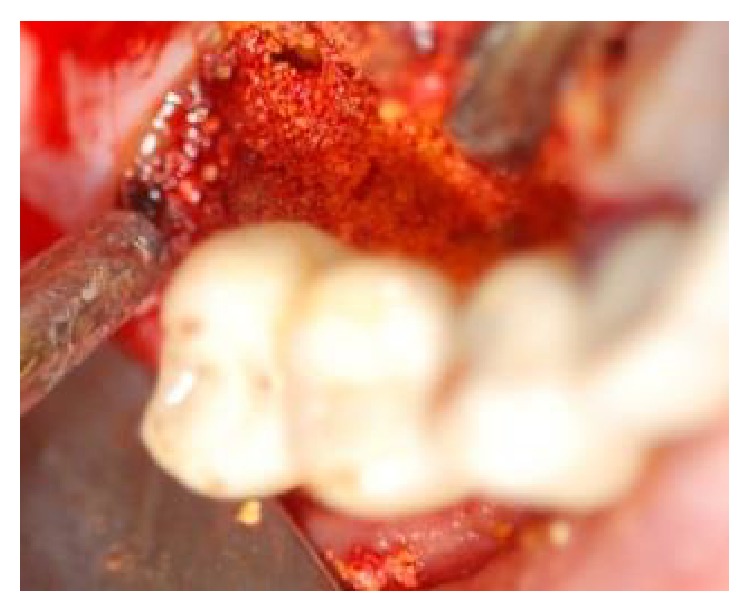
Following decontamination of the implant surface, a mixture of biphasic calcium sulfate and inorganic bovine bone were applied to defect at the buccal and palatal location (without a membrane).

**Figure 8:**
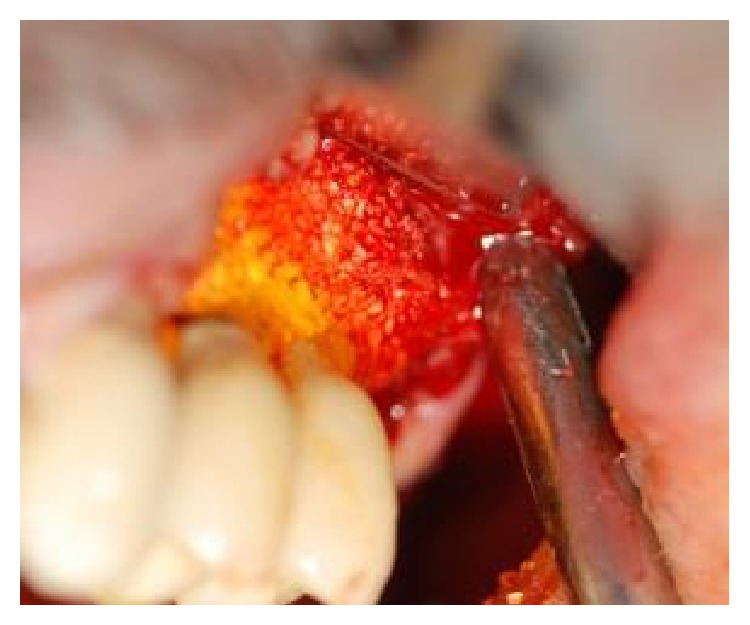
Following decontamination of the implant surface, a mixture of biphasic calcium sulfate and inorganic bovine bone were applied to defect at the buccal and palatal location (without a membrane).

**Figure 9:**
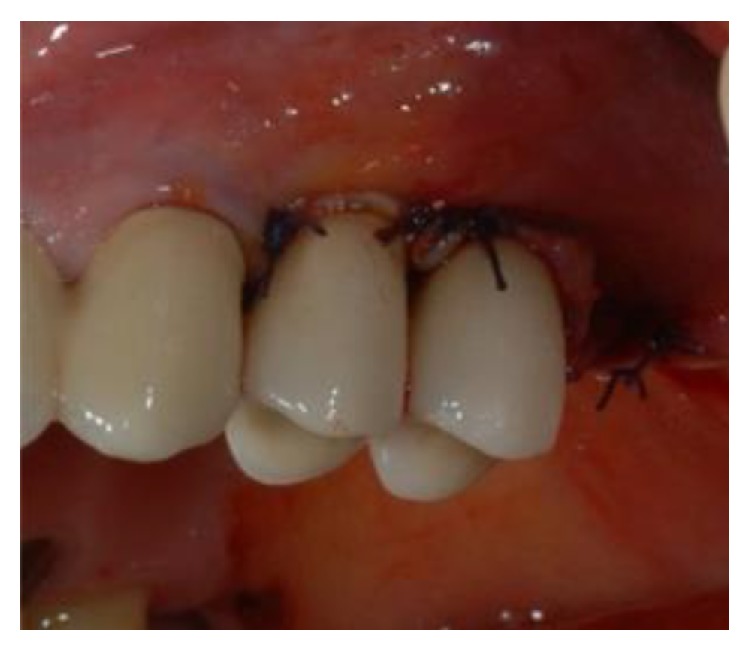
The mucoperiosteal flap was repositioned to ensure transmucosal healing and proper wound closure.

**Figure 10:**
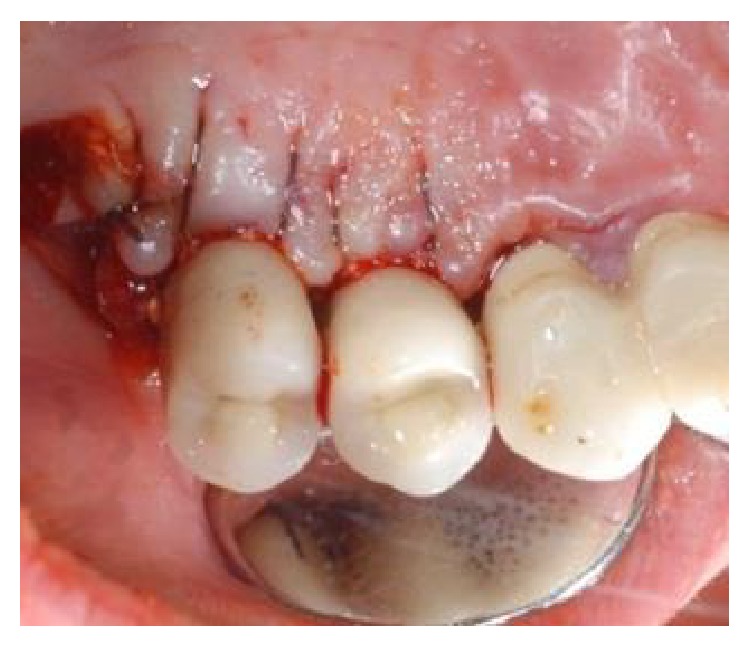
The mucoperiosteal flap was repositioned to ensure transmucosal healing and proper wound closure.

**Figure 11:**
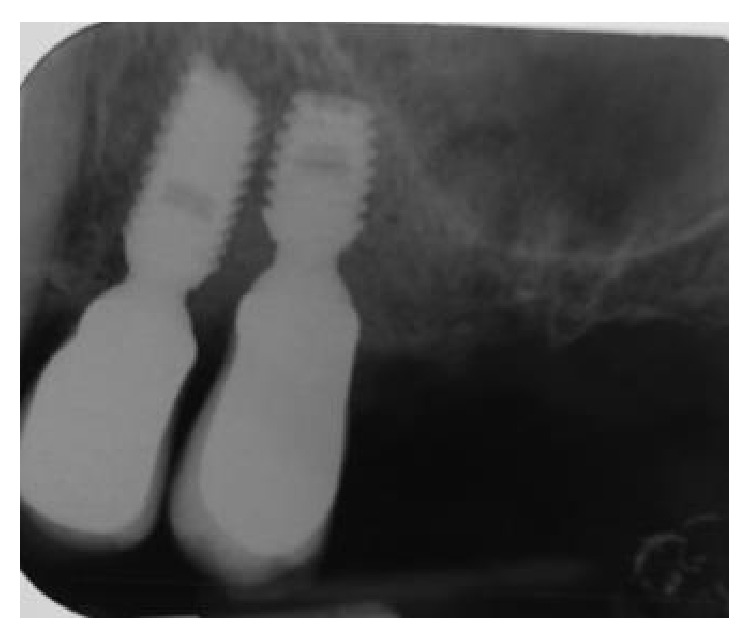
Postoperative radiograph indicating complete filling of the peri-implant defect.

After the second round of surface decontamination had concluded, bone defects were filled with a composite graft that was created by mixing the inorganic portion of bovine bone (Bio-Oss) with a synthetic biphasic calcium sulfate material (BondBone). Bio-Oss (0.5 mg) and BondBone (0.5 g) were combined with Rifampicin (1 vial, Sanofi-Aventis) to produce the composite. Because the composite bone graft possesses the ability to harden in the presence of blood and saliva, we decided to forgo the use of graft retaining membranes (Figures 7 and 8). The flap was then mobilized and advanced in order to obtain a primary tension-free closure (Figures 9 and 10). Following the procedure, postoperative radiographs were taken to evaluate the level of the defect filling (Figure 11).

Postoperative care included a 0.12% chlorhexidine + 0.05% cetylpyridinium chloride (CPC) rinse (GUM Paroex, Sunstar Suisse S.A.) twice daily for 2 weeks, 1 g of amoxicillin every 12 hours for 7 days, and 800 mg of ibuprofen as needed for pain. Following the procedure the patient was instructed to abstain from brushing for two weeks and flossing for one month.

The patient was evaluated one week following the procedure. At two weeks, the patient was reevaluated and the sutures were removed. Four weeks after procedure the patient underwent surgical area debridement and home oral hygiene techniques were reinforced. The patient was placed on an 8–12 week recall schedule until the completion of treatment (2 years), during which time periapical radiographs were taken every 6 months.

## 3. Results

Measurements derived from clinical observations are summarized in Table 1 and represent the mean of four sampling sites surrounding each implant (i.e. buccal, lingual/palatal, mesial, and distal) both immediately before surgery (baseline) and at the 2-year time point.

Bone level changes and percentage of bone fill were measured using scanning intraoral radiographs with parallel technique, using Rinn centering devices (Rinn XCP Posterior Aiming Ring-Yellow, Dentsply, Elgin, IL). First bone-to-implant contact changes were assessed as described by Urdaneta et al. (2010) [18]. Implant measurements taken from radiographs were calibrated to actual implant lengths by using ImageJ to calculate the pixel/mm ratio of radiographs taken at baseline and after 2 years.

**Figure 12:**
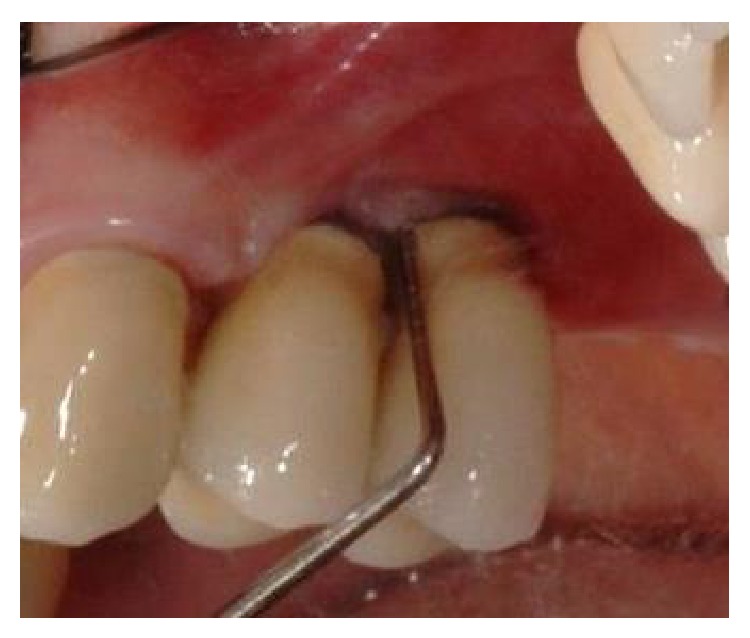
One-year after procedure, an absence of bleeding and reduced probing depth was observed.

**Figure 13:**
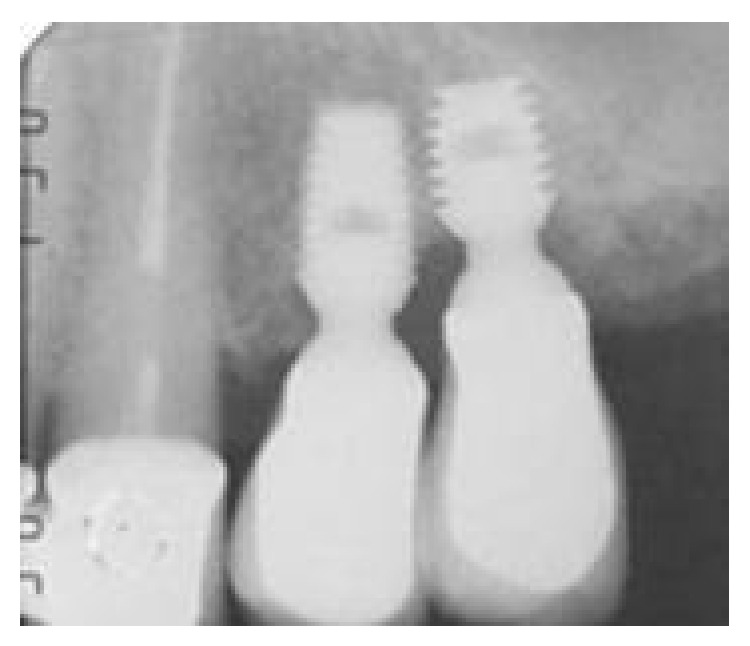
One-year postoperative radiographs depicting radiopacity at the location of the defect.

**Figure 14:**
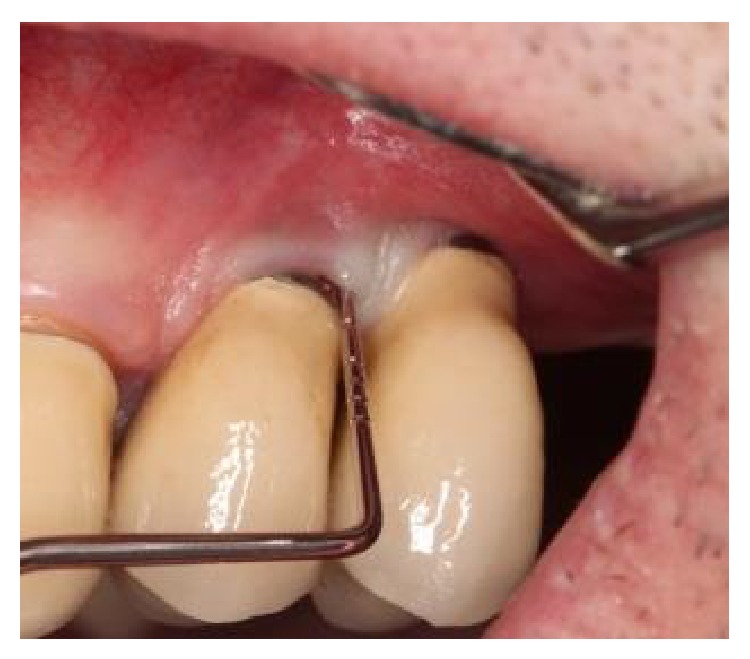
Two-year postoperative clinical examination revealed health hard and soft tissues with no bleeding and reduced probing depth (buccal and lingual/palatal probing shown).

**Figure 15:**
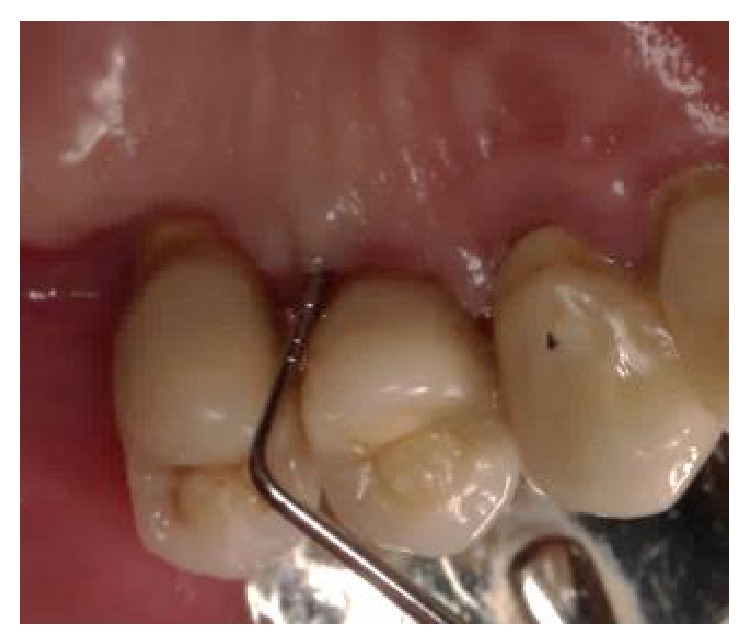
Two-year postoperative clinical examination revealed health hard and soft tissues with no bleeding and reduced probing depth (buccal and lingual/palatal probing shown).

After an uneventful healing period of one year, clinical evaluations revealed healthy peri-implant hard and soft tissues (Figures 12 and 13). Two years after procedure, a complete absence of bleeding upon probing along with physiological probing was observed (Figures 14 and 15). A slight recession of the soft tissues without a change in the height of keratinized tissue was also observed (Table 1).

**Figure 16:**
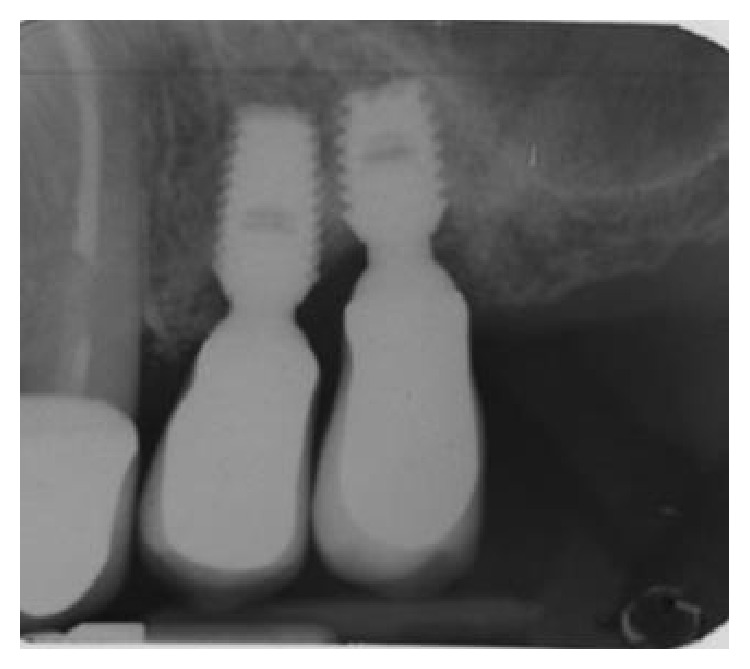
Two-year postoperative radiographs depicting radiopacity at the location of the defect, showing a near complete regeneration of the missing bone.

Radiograph measurements reflecting bone level changes and the percentage of bone fill are summarized in Table 2. Radiographs revealed that the initial bony defect had been almost completely regenerated (Figure 16), and increases in radiographic first bone-to-implant contact were observed at both the mesial (2.9 mm and 6.7 mm at numbers 12 and 13, resp.) and distal regions (8.0 mm and 5.6 mm at numbers 12 and 13, resp.).

## 4. Discussion

Peri-implantitis is responsible for the majority of implant failures [3, 14, 17]. Defined as an irreversible condition with a relatively high—and possibly underreported—incidence rate, the prevention and treatment of peri-implantitis should be of the utmost concern to every clinician [3–5, 13].

Many factors come into play when choosing an appropriate treatment for peri-implantitis. Overall patient health, location of the defect, and progression of the disease are all factors that should be considered. When probing depths exceed 5 mm, bleeding on probing occurs, and conventional nonsurgical options have been exhausted; then surgical intervention involving open debridement with resective or regenerative therapy should be performed [19–21].

We chose to pursue a novel course of treatment that utilized a topical desiccant and air powder abrasion to decontaminate the afflicted site, followed by grafting a mixture of biphasic calcium sulfate and inorganic bovine bone. The use of air powder abrasion during open flap surgical procedures has been shown to be an efficient decontamination measure both* in vitro* and* in vivo* [22–28]. And surfaces treated with air powder abrasives do not significantly affect the viability of human gingival fibroblasts and osteosarcoma cells,* in vitro* [29, 30]. Despite the efficacious nature of air powder abrasion as highlighted in the relevant literature, defect and implant morphology can potentially diminish its effectiveness (especially in cases of narrow defects and around implant threads) [22–28, 31]. In addition, the use of adjunctive antibiotics or antiseptics to air powder abrasion has yielded favorable outcomes [32]. Bearing this in mind, we chose to use the desiccating agent HYBENX as an adjunct to air powder abrasion.

HYBENX—an extremely hygroscopic solution comprised of hydroxymethoxybenzenesulfonic and hydroxybenzenesulfonic acid isomers, sulfuric acid, and water—has been used in the treatment of recurrent aphthous stomatitis [33]. The use of HYBENX was pursued in an attempt to boost the efficacy of our decontamination procedure, by denaturing adherence proteins used by bacteria in narrow defects and on implant threads, allowing them to be more easily rinsed away [31].

Subsequent to decontamination, we grafted a combination of inorganic bovine bone with biphasic calcium sulfate, in the absence of a membrane. Membranes (in the context of implant surgeries) serve as scaffolds to guide bone growth as well as barriers to soft tissue invasion [34, 35]. Owing to the fact that the novel composite we used possesses the ability to harden in the presence of saliva and blood (and in light of a report indicating no significant difference between bone fill levels when comparing resorbable membranes to bone substitute alone), we decided to forgo the use of membranes [36].

## 5. Conclusion

The absence of morbidity and remarkably uneventful healing period our patient exhibited suggest that the technique described here may represent a successful procedure in the treatment of peri-implantitis. Clinical and radiographic evidence presented here corroborate the efficacy of this procedure. Based on the outcomes of this report, future work using this novel decontamination and bone grafting procedure should be considered.

## Figures and Tables

**Table 1:** Postsurgical clinical observations taken at baseline and 2 years. Baseline values were obtained immediately before surgery. Results are expressed as Mean ± SD and are the average values of the four areas investigated. BOP = bleeding on probing, PD = probing depth, MR = mucosal recession, CAL = clinical attachment level, and KM = keratinized mucosa width.

Implant site (tooth number)	PPD (mm)		BOP (%)		MR		KM		CAL	
	Baseline	2 years	Baseline	2 years	Baseline	2 years	Baseline	2 years	Baseline	2 years
12	7.0 ± 1.8	2.7 ± 0.5	100	0	1.2 ± 0.7	1.0 ± 0.9	2.3 ± 0.6	2.4 ± 0.6	8.2 ± 2.3	3.7 ± 0.8
13	8.3 ± 1.0	2.8 ± 0.4	100	0	0.8 ± 0.7	1.4 ± 0.5	1.0 ± 1.0	1.0 ± 1.0	9.2 ± 1.2	4.1 ± 0.8

**Table 2:** Radiographic observations observed at baseline after 2 years and expressed as variations after 2 years. Baseline values were obtained immediately before surgery. Δ = changes of values compared with baseline after 2-year follow-up period. r-BF = the percentage of radiographic bone fill of the defect at 2 years.

Implant site (tooth number)	First bone-to-implant contact (FBIC)						
	Mesial (mm)			Distal (mm)			r-BF (%)
	Baseline	2 years	Δ	Baseline	2 years	Δ	
12	−3.0 mm	−0.1 mm	+2.9 mm	−8.1 mm	−0.1 mm	+8.0 mm	93.0%
13	−6.9 mm	−0.2 mm	+6.7 mm	−5.8 mm	−0.2 mm	+5.6 mm	91.6%
